# Toward the identification of a type I toxin-antitoxin system in the plasmid DNA of dairy *Lactobacillus rhamnosus*

**DOI:** 10.1038/s41598-017-12218-5

**Published:** 2017-09-21

**Authors:** Claudia Folli, Alessia Levante, Riccardo Percudani, Davide Amidani, Stefania Bottazzi, Alberto Ferrari, Claudio Rivetti, Erasmo Neviani, Camilla Lazzi

**Affiliations:** 1 Department of Food and Drug, University of Parma, 43124 Parma, Italy; 2 Interdepartmental Center SITEIA.PARMA, University of Parma, 43124 Parma, Italy; 3 Department of Chemistry, Life Sciences and Environmental Sustainability, University of Parma, 43124 Parma, Italy

## Abstract

Plasmids carry genes that give bacteria beneficial traits and allow them to survive in competitive environments. In many cases, they also harbor toxin-antitoxin (TA) systems necessary for plasmid maintenance. TA systems are generally characterized by a stable “toxin”, a protein or peptide capable of killing the cell upon plasmid loss and by an unstable “antitoxin”, a protein or a non-coding RNA that inhibits toxin activity. Here we report data toward the identification of a RNA-regulated TA system in the plasmid DNA of *L*. *rhamnosus* isolated from cheese. The proposed TA system comprises two convergently transcribed RNAs: a toxin RNA encoding a 29 amino acid peptide named Lpt and an antitoxin non-coding RNA. Both toxin and antitoxin RNAs resulted upregulated under conditions mimicking cheese ripening. The toxicity of the Lpt peptide was demonstrated in *E*. *coli* by cloning the Lpt ORF under the control of an inducible promoter. Bioinformatics screening of the bacterial nucleotide database, shows that regions homologous to the Lpt TA locus are widely distributed in the *Lactobacillus* genus, particularly within the *L*. *casei* group, suggesting a relevant role of TA systems in plasmid maintenance of cheese microbiota.

## Introduction

*Lactobacillus rhamnosus* is a non-starter lactic acid bacterium that plays a significant role during cheese ripening, contributing to the formation of flavor. In long-ripened cheeses it persists throughout the whole ripening time, due to its ability to adapt to changing environmental conditions, however the metabolic response of *L*. *rhamnosus* to different ecosystems is still poorly understood.

Fermented foods like cheese represent challenging environments for bacteria because the nutrients are often exhausted and waste products are abundant. In addition, other factors such as moisture content, salt concentration, pH and oxygen concentration can affect survival, growth and metabolism of the cheese microbiota^1^. Several studies have been carried out to better comprehend the bacterial metabolic response under these adverse conditions, particularly with the aim to identify biochemical pathways that promote bacterial survival and growth within the cheese habitat^2^. Recently we identified a set of genes related to the growth of *L*. *rhamnosus* in a cheese-like medium by cDNA-amplified fragment length polymorphism (cDNA-AFLP), a sensitive transcriptomic technologies for gene expression analyses^3^. In this study, the upregulation of a transcript related to a *Lactobacillus casei* plasmid was also reported. To date, more than 20 species of *Lactobacillus* containing plasmids have been identified and over 400 lactic acid bacteria (LAB) plasmids have been isolated and characterized^4^. Among these, only four plasmids have been isolated from *L*. *rhamnosus* and their sequence is available in GenBank (pLR001, GenBank accession number CP001155; pLR002 GenBank accession number CP001156; pLC1 GenBank accession number FM179324; plasmid from strain BFE5264 GenBank accession number CP014202). Plasmids often carry genes that are essential for survival under adverse conditions^5^. In particular, LAB plasmids encode traits related to phages or antibiotics resistance, lactose catabolism and production of proteolytic enzymes or bacteriocins^4^. LAB plasmids may also harbor genes encoding toxin-antitoxin (TA) systems necessary for plasmid maintenance^6–11^, a phenomenon known as plasmid addiction or post-segregational killing.

Plasmid TA loci are two-component systems made of a stable “toxin”, capable of killing the cell, and an unstable “antitoxin” that inhibits the toxin activity^12^. When a plasmid-free variant is produced, owing to a replication error or to defects in plasmid maintenance, a rapid depletion of the antitoxin occurs in newborn plasmid-free cells. Under these conditions, the stable toxin inherited from the mother cell no longer neutralized by the antitoxin, causes cell death^13^. Homologous systems have also been found in bacteria chromosomes as part of integrated mobile elements^14,15^. However, newly identified TA loci of the bacterial chromosomes do not show homology with mobile genetic elements^14^, suggesting that TA systems might have different biological functions.

To date, at least six types of TA systems with different genetic architectures and regulatory activities have been identified^16,17^. Among these, type I TA system is characterized by a toxin peptide and an antitoxin RNA capable of interfering with the toxin mRNA translation either by preventing ribosome binding or by promoting mRNA degradation. Studies focused on type I TA systems are limited, probably because the small size of the toxin peptide and the complex prediction of small RNAs make their recognition difficult^18^.

In this work we report data in support of the presence of a type I TA system in the plasmid DNA of two different strains of. *L*. *rhamnosus* isolated from Parmigiano-Reggiano cheese. The proposed TA system includes a 29 amino acid hydrophobic toxin named Lpt (*Lactobacillus* plasmid toxin) and a 74 nucleotides antitoxin RNA. The antitoxin RNA harbours a 24 nt sequence complementary to a region encompassing the start codon of the toxin mRNA. Both toxin and antitoxin RNA are upregulated in a cheese-like medium, suggesting a possible role under stress conditions. Moreover, bioinformatics analysis shows that regions homologous to the proposed TA locus are widely distributed in plasmids harbored by the *L*. *casei* group (*L*. *rhamnosus*, *L*. *casei* and *L*. *paracasei*) and by *L*. *brevis*, suggesting a relevant function of TA systems in the *Lactobacillus* genus.

## Results

### Identification of the putative TA locus

**Figure 1:**
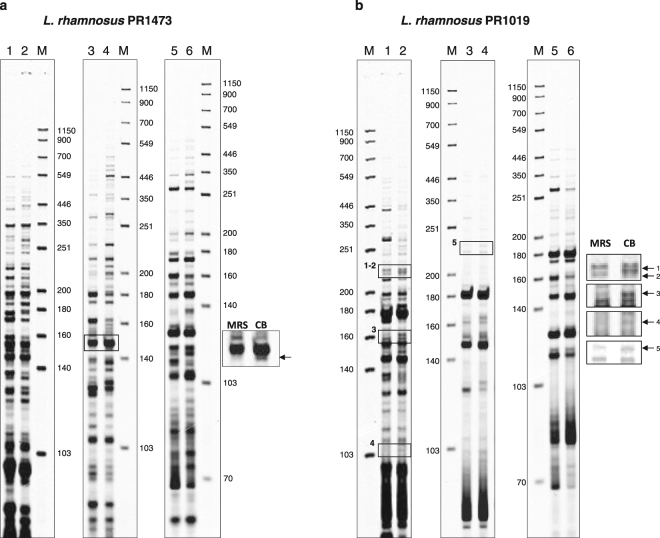
Electrophoretic patterns of cDNA-AFLP experiments conducted on *L*. *rhamnosus* strains PR1473 (**a**) and PR1019^3^ (**b**) grown in MRS or CB medium. Lane M, IRDye700 sizing standard. Lanes 1, 3 and 5, MRS medium with the following primer combinations: lane 1, EcoRI-AC/MseI-AT; lane 3, EcoRI-AT/MseI-AC; lane 5, EcoRI-AT/MseI-AT. Lanes 2, 4 and 6, CB medium with the following primer combinations: lane 2, EcoRI-AC/MseI-AT; lane 4, EcoRI-AT/MseI-AC; lane 6, EcoRI-AT/MseI-AT. The overexpressed amplified fragments corresponding to the plasmid sequence are boxed and magnified on the right side of each gel. The full length images of the gels are reported in Fig. S1.

Figure 1a shows the transcriptomic profile of *L*. *rhamnosus* PR1473 grown in MRS and Cheese Broth (CB) media evaluated by using cDNA-AFLP with different primer sets. By comparing these results with those previously observed for the strain PR1019^3^ (Fig. 1b), a sequence overexpressed in CB medium in both strains was identified. This sequence corresponds to one differentially amplified fragment in PR1473 and to five differentially amplified fragments in PR1019 (Fig. 1a,b and Fig. S1).

**Figure 2:**
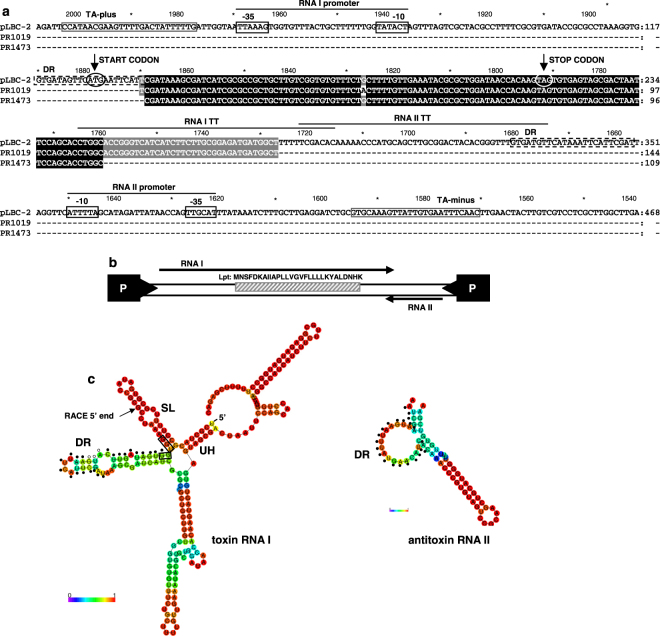
Structure of the putative toxin–antitoxin locus. (**a**) Alignment between the plasmid sequences overexpressed in *L*. *rhamnosus* PR1473 and PR1019 identified by cDNA-AFLP and homologous DNA sequence of plasmid pLBPC-2 from *L*. *paracasei* (GenBank accession number AP012543 range 2009–1541). The predicted elements of the identified TA locus are: black lines, RNA I and RNA II promoters and corresponding transcription terminators (TT); dashed black boxes, direct repeat regions (DR); black arrows, start and stop codons of the toxin peptide; gray boxes, primers TA-plus and TA-minus employed to amplify the complete TA system region. (**b**) Schematic representation of the Lpt TA locus showing the two convergently transcribed RNA I and RNA II (black arrows) and the corresponding promoters (P boxes). The shaded box represents the toxin coding region with the peptide sequence written above. (**c**) Secondary structures of RNA I (toxin) and RNA II (antitoxin) predicted with the RNAfold WebServer. The 5′ end of the toxin RNA identified by RACE experiments is indicated with an arrow. DR, direct repeat region; UH, upstream helix; SL stem-loop. On toxin and antitoxin RNAs, dots indicate DR sequences. On toxin RNA black boxes indicate the RBS region and white dots the Lpt start codon. RNA nucleotides are colored by base-pairing or unpairing probabilities.

By searching the DNA database with the Blastn program using the overexpressed transcript from the strain PR1473 as a query, we retrieved two plasmid sequences belonging to strains of the *L*. *casei* group. In particular, high similarity was found with the plasmid pLBPC-2 from *L*. *paracase*i subsp. *paracasei* JCM8130 and with the plasmid pNCD0151 from *L*. *casei* (100% and 99% identity over 109 nucleotides, respectively). These homologous sequences are annotated in the database as non-coding DNA regions and no significant similarity was found by searching the protein database with the Blastx program^19^. However, a search in the Conserved Domain (CD) database^20^ revealed a marginally significant similarity (E = 8 × 10^−3^) between an open reading frame (ORF) encoding a 29 amino acids peptide and the *faecalis* plasmid stabilization toxin (Fst) domain. A sensitive homology search with the HHPRED program^21^, using the peptide sequence of the hypothetical ORF as a query, resulted in the identification of several bacterial toxins. The highest similarity (E = 2 × 10^−17^) was found with Fst, a type I TA system toxin, which was first identified in the pAD1 plasmid of *Enterococcus faecalis* ^22,23^. Based on these hints we analyzed a larger region of the pLBPC-2 plasmid in order to assess the existence of a complete regulatory region characterizing the toxin-antitoxin mechanism. By means of bioinformatics tools, two small convergently transcribed RNAs (RNA I and RNA II), typical of type I TA systems^10^, were identified (Fig. 2a,b). RNA I encodes a 29 amino acids hydrophobic peptide with a putative role of toxin (Fig. 2b) that we named *Lactobacillus* plasmid toxin (Lpt). Interestingly enough, the plasmid sequences overexpressed in the two *L*. *rhamnosus* strains in CB medium (Fig. 1), correspond to a portion of RNA I (Fig. 2a).

Both RNA I and RNA II contain a 24 nt highly complementary sequence (DR in Fig. 2a) that may be involved in molecular interaction. Furthermore, as shown in Fig. 2c, secondary structure prediction of RNA I and RNA II suggests folds which are similar to those proposed for toxin and antitoxin RNAs in the Fst TA system^22,23^. In this system, the upstream helix (UH) located at the 5′ end of toxin RNA is essential to ensure RNA stability^24^, while the flanking stem loop region (SL), including the ribosomal binding site (RBS) sequence, is implicated in an intramolecular mechanism of translation repression^25^. In analogy with the Fst TA system, in the structural model of the Lpt-encoding RNA (Fig. 2c), a putative UH is identified at the 5′ end, followed by a SL region that includes most of the RBS sequence. The RNA I DR sequence includes the Lpt start codon and is located in a paired region characterized by a low pairing stability, whereas the antitoxin RNA II DR sequence is mainly located in a single stranded region (Fig. 2c). Therefore, by analogy with the Fst TA system, we suggest that the Lpt toxin synthesis is controlled by two different mechanism of translation inhibition: an intramolecular regulation involving SL region capable of targeting the RBS sequence, and an intermolecular mechanism mediated by the interaction between complementary DR sequences of RNA I and RNA II.

### Characterization of the putative TA locus in L. rhamnosus

**Figure 3:**
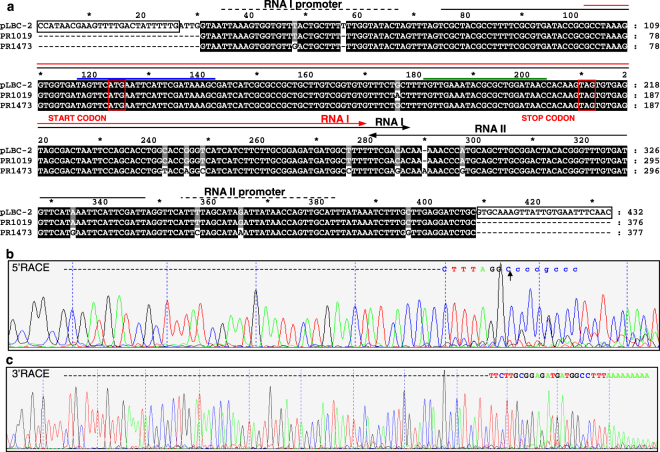
Characterization of *L*. *rhamnosus* RNA I and RNA II sequence elements. (**a**) Sequence alignment of the TA region amplified from *L*. *rhamnosus* PR1473, *L*. *rhamnosus* PR1019 and the homologous sequence of *L*. *paracasei* pLBPC-2 plasmid. TA-plus and TA-minus primers used in PCR (black boxes); predicted RNA I and RNA II transcripts (black arrows); experimentally determined RNA I transcript (red arrow); predicted promoter regions (black dashed lines); start and stop codons of the toxin-coding region on RNA I (red boxes); sequence-specific primers used in 3′ RACE (blue line) and 5′ RACE (green line) experiments. (**b**) *L*. *rhamnosus* PR1019 sequencing profile of the amplified fragment obtained by 5′ RACE experiments. The 5′ end of the toxin RNA is in uppercase characters, the primer sequence is in lowercase characters and the black arrow points to the 5′ end nucleotide (see also Fig. S3). (**c**) *L*. *rhamnosus* PR1473 sequencing profile of the amplified fragment obtained by 3′ RACE experiments. The 3′ end of the toxin RNA is in uppercase characters.

The region containing the TA locus was PCR-amplified from plasmid DNA extracted from *L*. *rhamnosus* PR1473 and *L*. *rhamnosus* PR1019 grown in MRS or CB medium by using primers designed on the pLBPC-2 sequence (Fig. 3a, Table S1). The results were positive for all the conditions analyzed (Fig. S2), suggesting that the TA system is located on a plasmid which is retained during growth under different conditions.

The DNA regions amplified from the two different strains were sequenced and compared to the plasmid pLBPC-2 deposited in GenBank (Fig. 3a). In the sequence we found the C-terminal part of a protein annotated as “Initiator Replication protein” (RepB), involved in plasmid replication, further supporting the hypothesis that the TA locus is located on a plasmid. The sequence from PR1473 strain shares a 97% identity with the sequence from PR1019 strain and with pLBPC-2 plasmid, while the sequence from PR1019 strain shares an identity of 99% with pLBPC-2 plasmid. The Lpt toxin coding region is identical among the three compared sequences with the only exception of a conservative single nucleotide substitution in PR1019 strain (Fig. 3a). In order to experimentally verify the length of the *in-silico* predicted coding transcript RNA I, RACE experiments were carried out starting from total enriched RNA extracted from PR1473 and PR1019 strains grown in CB medium. RACE identified the 5′ end of the RNA I transcript 27 nucleotides downstream of the predicted transcription starting site (Fig. 3a,b; Fig. S3a,c) and the 3′ end 8 nucleotides upstream of the predicted transcription termination site (Fig. 3a,c; Fig. S3b). The experimentally identified RNA I molecule is thus shorter than the predicted molecule and possesses a transcription starting site located at a non-conventional distance from the putative promoter sequence. It should also be noted that there is clearly ambiguity in the 5′ RACE sequence beyond the mapped site, suggesting that multiple products are present. Based on these observations, we speculate that the RNA I transcript may be processed by specific cleavage at the 5′ end. Processing of the toxin RNA has been previously proposed for the Fst TA system as a possible intramolecular mechanism of translation activation, however, the processed molecule was not identified^14^.

### Detection of RNA I and RNA II by quantitative reverse transcription PCR

**Figure 4:**
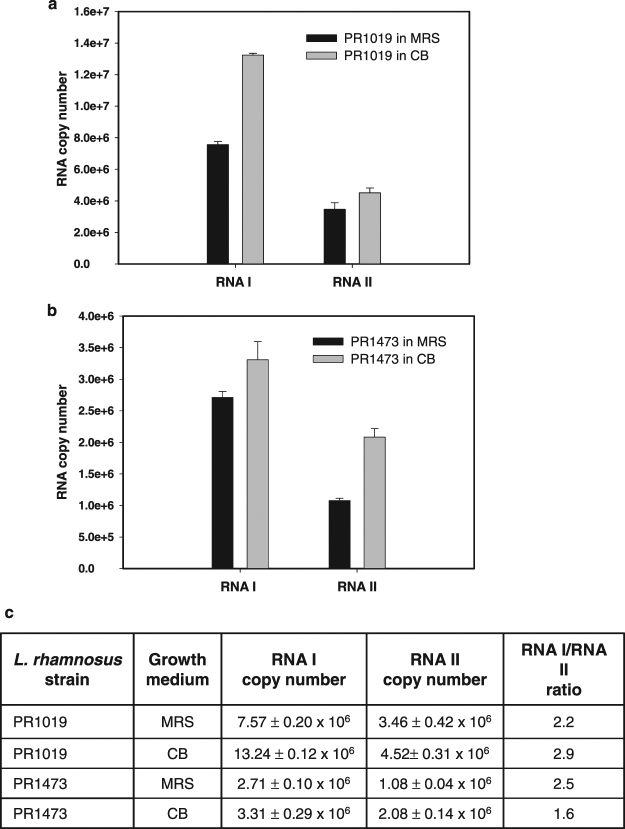
Detection of RNA I and RNA II by qRT PCR. (**a**) Absolute quantification of RNA I and RNA II in *L*. *rhamnosus* PR1019. MRS medium (black bars); CB medium (grey bars). (**b**) Absolute quantification of RNA I and RNA II in *L*. *rhamnosus* PR1473. MRS medium (black bars); CB medium (grey bars). The error bars represent the standard deviation of the mean value from three independent experiments. (**c**) Summary table with RNA I and RNA II copy number values and corresponding ratio obtained for each strain in MRS and CB media.

To confirm the reliability of cDNA-AFLP results, a quantitative reverse transcription PCR (qRT PCR) was carried out to evaluate the expression level of the toxin mRNA I in *L*. *rhamnosus* PR1473 and PR1019 strains grown in MRS or CB medium (Fig. 4). Quantification of the antitoxin RNA II was evaluated under the same conditions (Fig. 4). For both strains, the copy number of RNA I and RNA II resulted higher in CB medium in comparison with MRS medium (p < 0.05) and the estimated copy number of RNA I was higher than RNA II in all the analyzed samples (Fig. 4). The computed ratio between toxin and antitoxin RNAs for growths in MRS medium was 2.2 and 2.5 for PR1019 and PR1473 strains, respectively (data not statistically different, not shown), while in CB medium, the computed ratio was 2.9 and 1.6 for PR1019 and PR1473 strains, respectively (Fig. 4c).

In addition, detection of the toxin RNA was carried out by qRT PCR on RNA extracted from two samples of Parmigiano Reggiano cheese, at 6 and 12 months of ripening, when *L*. *rhamnosus* represents the predominant species^26^. The relative expression of Lpt mRNA was confirmed also *in situ*, with slight variations between the two samples (Fig. S4).

### Toxicity in *E. coli*

**Figure 5:**
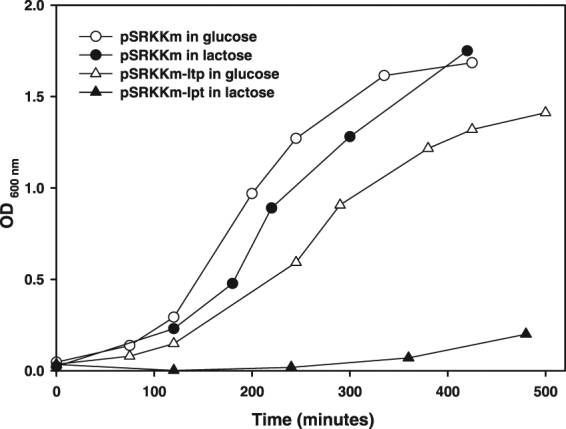
Lpt toxicity in *E*. *coli.* Growth assays of *E*. *coli* DH10bT1R transformed with the empty vector pSRKKm (circles) or with the recombinant pSRKKm-lpt vector (triangles), in LB medium supplemented with lactose (black symbols) or glucose (white symbols).

To verify the toxicity of the predicted Lpt peptide, the corresponding cDNA was cloned in the lactose-inducible expression vector pSRKKm^27^ and the recombinant plasmid was used to transform *E*. *coli* DH10bT1R. As shown in Fig. 5, standard growth curves were obtained for cells transformed with an empty pSRKKm vector in the presence of either glucose (white circles) or lactose (black circles). When *E*. *coli* was transformed with recombinant plasmid pSRKKm-lpt, the growth in the presence of glucose (white triangles) shows a small delay, probably due to basal transcription of the *lac* promoter. In the presence of lactose (black triangles), a condition that induces the expression of the Lpt peptide, growth was strongly inhibited. This result clearly demonstrates that the predicted Lpt peptide has a toxic effect on *E*. *coli*.

### Promoter validation by AFM mapping of RNAP binding

**Figure 6:**
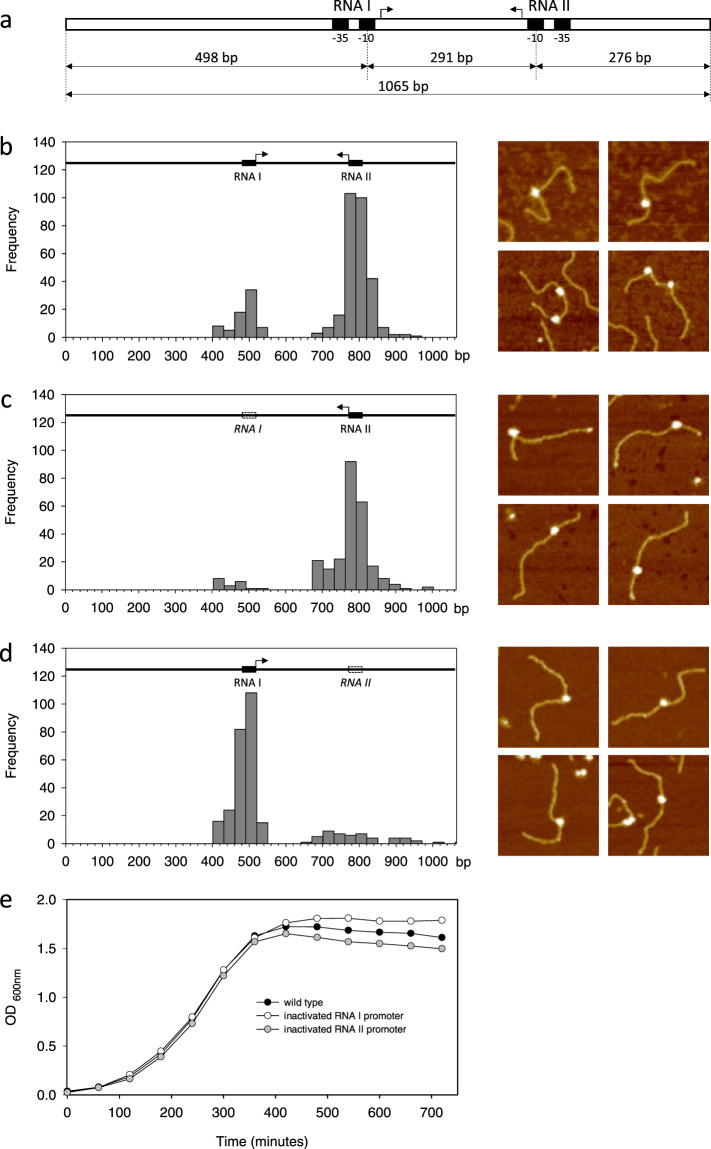
Promoter mapping by atomic force microscopy. (**a**) Schematic representation of the 1065 bp long DNA fragment used in AFM experiments. Position of the promoters from the DNA ends is given with respect to the centre of the −10 hexamer. (**b**–**d**) Distribution of the RNAP position along the DNA template determined by contour length measurements of RNAP-DNA complexes imaged by AFM. The DNA template used is schematized at the top of each panel and representative complexes are shown on the right side. (**b**) *wt* RNA I and *wt* RNA II promoters; (**c**) inactivated RNA I and *wt* RNA II promoters; (**d**) *wt* RNA I and inactivated RNA II promoters. (**e**) Growth assays of *E*.*coli* XL1 Blue transformed with pGEM-TA harboring *wt* RNA I and *wt* RNA II promoters (black circles), inactivated RNA I and *wt* RNA II promoters (white circles) and *wt* RNA I and inactivated RNA II promoters (grey circles).

To verify that the predicted promoter sequences of RNA I and RNA II are actual promoters, we employed Atomic Force Microscopy (AFM) to map the position of RNA polymerase (RNAP) bound along a DNA template of known sequence. First, the 431 bp DNA sequence carrying the entire TA system was cloned into pGEM T-easy to obtain plasmid pGEM-TA (see Methods). Using this construct and primers mapping on the vector DNA, a linear DNA fragment of 1065 bp was obtained by PCR. Within this fragment, the RNA I promoter is located 498 bp from the upstream DNA end, whereas the RNA II promoter is located at 276 bp from the upstream DNA end (Fig. 6a). Promoter complexes were assembled with *E*. *coli* σ^70^-RNAP, deposited onto freshly-cleaved mica and imaged in air by AFM as described in the Methods. Representative full-scan AFM images of the complexes are reported in Fig. S5. Next, AFM images were processed to measure the DNA contour length and to determine the position of bound RNA polymerases relative to the DNA ends. As shown in Fig. 6b, RNAP preferentially binds the DNA template at two positions which correspond well with the position of the predicted RNA I and RNA II promoters. RNAP-DNA complexes were classified as “specific promoter complexes” when they were within ± 40 bp from the −10 hexamer of the corresponding promoter. Among all the complexes scored, we could estimate that 66% are formed at RNA II promoter, 17% are formed at the RNA I promoter while the rest are nonspecific complexes. DNA templates with both promoters occupied by an RNAP were observed only in few cases.

To confirm that the observed complexes are formed at the predicted promoters, the promoters were alternatively inactivated by introducing three point mutations into the corresponding −10 hexamer^28,29^ and their effect on the position distribution of RNAP-DNA complexes was analyzed. Figure 6c shows that the inactivation of the RNA I promoter drastically reduces the number of RNAP bound near the center of the DNA template where this promoter is located. As expected, RNAP binding to the *wt* RNA II promoter of this template remains unaffected. Likewise, the inactivation of the RNA II promoter drastically reduces the number of RNAP bound in the template region corresponding to this promoter (Fig. 6d). Interestingly, with this DNA template we observed a significant increase of the number of complexes formed at the *wt* RNA I promoter. These results confirm that the predicted RNA I and RNA II promoters are actual promoters, capable of binding *E*. *coli* σ^70^-RNAP. Based on the relative promoter occupancy, these results further suggest that the antitoxin promoter is about four times stronger than the toxin promoter, at least in terms of RNAP recruitment.

### *E. coli* growth assays

To evaluate the *in vivo* activity of the predicted RNA I (toxin) and RNA II (antitoxin) promoters, we transformed *E*. *coli* with plasmids containing the entire TA sequence either in the wild type form or with the RNA I and RNA II promoters alternatively inactivated. In these constructs, transcription of RNA I and RNA II is under the control of their *wt* or mutated natural promoters.

Figure 6e shows that in all cases the transformed cells reach an OD_600_ of about 1.7–1.8 in 6 hours. The OD value remains stable in the next 5 hours for *E*. *coli* transformed with plasmid containing the inactivated RNA I promoter (white circles). Conversely, the OD_600_ values of *E*. *coli* transformed with plasmid containing the wild type sequence (black circles) or the inactivated RNA II promoter (grey circles) decrease slowly but constantly in the next 5 hours, with a more consistent drop for cells with inactivated RNA II promoter. These *in vivo* results show specific *E*. *coli* phenotypes associated with RNA I or RNA II promoter inactivation and further validate the role of the predicted promoter sequences.

### Distribution of the Lpt TA locus in bacteria

**Figure 7:**
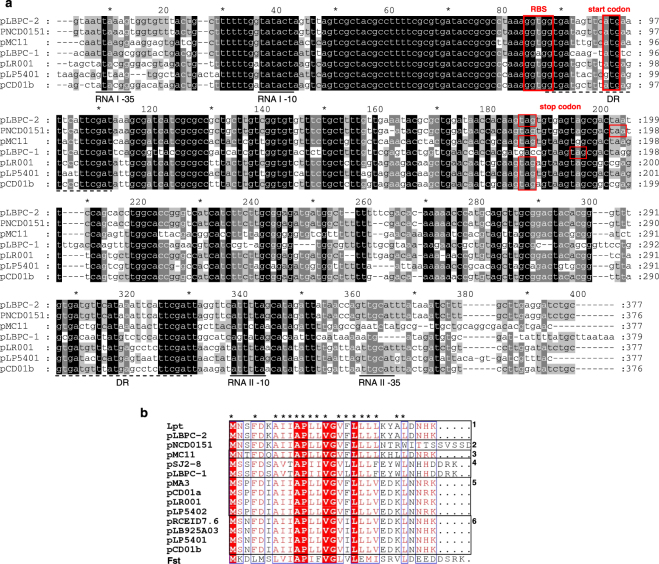
Alignment of TA sequence elements homologous to the *lpt* locus identified in plasmids of *Lactobacillus* genus (**a**) Representative sequence alignment of the putative TA systems. RNA I and RNA II promoters (black lines); direct repeat region DR (dashed black line); start codon, stop codon and RBS of RNA I (red boxes). Accession numbers and corresponding sequence intervals of the aligned TA loci are reported in Table 1. (**b**) Alignment of Lpt homologous peptides in *Lactobacillus* genus showing six different toxin peptides (numbered boxes) compared with the Fst toxin encoded by plasmid pAD1 of *E*. *faecalis*. Hydrophobic residues are marked with stars.

To assess the prevalence of Lpt TA system in bacteria, a screening of nucleotide bacterial sequences was performed by means of the Blastn program using the entire TA locus identified in *L*. *rhamnosus* PR1473 as a query. Interestingly enough, all the sequences with significant similarity are located on plasmids carried by bacteria belonging to the *Lactobacillus* genus. In particular, we were able to identify 6 plasmids isolated from *L*. *paracasei*, 3 isolated from *L*. *casei*, 2 from *L*. *brevis* and 1 from *L*. *rhamnosus* (Table 1). Comparison of the homologous plasmid regions containing the entire TA locus, revealed sequence identities ranging from 60% to 96% with the Lpt TA locus (Table 1 and Fig. 7a). Among these, plasmid pCD01 from *L*. *paracasei* is characterized by the presence of two TA loci (sequence a and b in Table 1), homologous to Lpt TA locus, located in non-adjacent DNA regions. Thus, the Lpt system seems to be specific for plasmids widespread among *Lactobacillus* genus, and in particular within *L*. *casei* group.

**Table 1:** Comparison between *lpt* locus of *L*. *rhamnosus* PR1473 and the homologous regions identified in plasmid of *Lactobacillus* species.

Plasmid	Organism	Identity	GeneBank	Sequence range
pLBPC-2	*L*. *paracasei* subsp. *paracasei* JCM 8130	96%	AP012543	1598–1974
pNCD0151	*L*. *casei*	96%	Z50861	3644–4019
pLR001	*L*. *rhamnosus* HN001	74%	CP001156	3317–3693
pCD01 (sequence a)	*L*. *paracasei* subsp. *paracasei* NFBC338	73%	AY662330	3749–4122
pLP5401	*L*. *paracasei*	71%	KC812101	3715–4091
pRCEID7.6	*L*. *casei* TISTR1341	71%	JN793951	1051–1425
pMC11	*L*. *casei* MCJ	70%	KF986324	10797–10421
pMA3	*L*. *paracasei* MA3	68%	EU255257	1614–1238
pCD01 (sequence b)	*L*. *paracasei* subsp. *paracasei* NFBC338	68%	AY662330	9015–9390
pLP5402	*L*. *paracasei*	67%	KC812102	5330–4955
pLB925A03	*L*. *brevis*	62%	AB370336	8351–8724
pSJ2-8	*L*. *paracasei* subsp. *paracasei*	62%	FM246455	10850–10479
pLBPC-1	*L*. *paracasei* subsp. *paracasei* JCM 8130	60%	AP012542	883–1261

In all plasmid sequences homologous to the Lpt TA locus, the RNA I and RNA II promoter and transcription termination regions here identified (Fig. 2a), are well conserved (Fig. 7a). The DR region found in pLBPC-2 (Fig. 2a) are present in all the compared plasmids, and are characterized by two highly conserved regions separated by a short plasmid-specific non-conserved sequence (Fig. 7a). Notably, the region corresponding to the predicted 5′ end of RNA I molecule, which includes the UH and the SL secondary structures (Fig. 2c), is highly conserved in all the plasmids (Fig. 7a). With regards to the translation signals, the start site of RNA I, represented by an ATG or GTG codon downstream of the RBS, are aligned in all the analyzed sequences. Conversely, the stop codon is aligned in 11 out of 14 sequences. In pSJ2–8 and pLBPC-1 it is located three codons downstream (Fig. 7a), while in pNCD0151, a single nucleotide deletion generates a frameshift leading to a peptide five amino acid longer (Fig. 7a,b). Lpt and its homologs toxin peptides reported in Fig. 7b, contain a number of amino acid residues ranging from 29 to 34 and share different sequence identities (from 38 to 100%). The comparison of the toxin sequences allows to classify the plasmids in six different groups (Fig. 7b), each characterized by a different toxin peptide; nevertheless, all peptides share highly conserved amino acids in specific positions (Fig. 7b), suggesting a structural or functional role for these residues.

The DNA sequence of the *lpt* locus contained a portion of a coding region corresponding to the C-terminal part of a peptide annotated as “Initiator Replication protein” (RepB). To evaluate the association between the TA locus and RepB, a synteny analysis^30^ of the different plasmids containing sequences homologous to Lpt was performed (Fig. S6). This analysis shows the presence of a region of conserved microsynteny encompassing the Lpt TA locus, a convergently transcribed *repB* gene and an upstream coding sequence transcribed in the same direction with respect to Lpt, annotated as Rep_B, a protein of unknown function with similarity to pfam04394 and cd04779 domains. Together with RepB, the Rep_B protein could be involved in plasmid replication, as proteins belonging to the same family are characterized as helix-turn-helix DNA binding proteins. The physical association with genes implicated in plasmid replication, already found for protein TA systems^31^, could favour the maintenance of Lpt TA locus.

## Discussion

The putative role of TA systems is generally referred to plasmid stability and maintenance but also other biological functions have been proposed. In particular, TA systems, defined one of the most versatile global regulatory system in bacteria^32^, are considered elements with the ability to cope with stress, to guard against DNA loss and to protect against phage invasion^33^. Type I toxin-antitoxin (TA) systems are characterized by a small toxin peptide and an antitoxin RNA capable of targeting the toxin mRNA. In this work we describe how the detection of an overexpressed RNA by transcriptomic analysis, lead us to the identification of a putative type I TA system in the plasmid DNA of two *L*. *rhamnosus* strains. Despite the overexpressed RNA shares significant sequence similarity with plasmid DNA of the *L*. *casei* group, detection of the TA system was complex. Firstly, the nucleotide regions with the highest identities, corresponding to plasmid pLBPC-2 of *L*. *paracasei* and plasmid pNCD0151 of *L*. *casei*, are annotated in the databases as non-coding sequences. Secondly, no homologs were found by searching the protein database with the Blastx program. Thirdly, although in the literature there are many reports describing toxin-antitoxin systems, only a few regard type I TA systems. This lack of information is probably due to the small size of the toxin peptide and to the challenging bioinformatics prediction of small RNAs. Known and new type I TA systems were recently identified in different bacterial lineages by using a computational approach based on PSI-Blast and tBlastn^18^, however, none of these referred to plasmids of the *Lactobacillus* genus. It was only thanks to a more sensitive tool for protein structure prediction (HHpred)^21^, that we could find a correlation between the overexpressed RNA in *L*. *rhamnosus* and the Fst protein family. Fst is a type I TA system, first identified on plasmid pAD1 from *E faecalis*, comprising two convergently transcribed small RNAs: RNA I coding for a toxin peptide and RNA II acting as an antitoxin^22^. Both RNA I and RNA II contain complementary DR sequences that are involved in the regulation of Fst expression. Because of similarities in the structural organization of the Lpt TA system with the Fst TA system, we hypothesize a common mechanism of action. Experiments conducted in *E*. *coli* have shown a strong inhibition of cell growth when the predicted Lpt toxin was expressed under the control of an inducible promoter. Furthermore, RACE experiments lead us to the identification of an RNA I transcript that was shorter than that predicted *in-silico*, suggesting that RNA I might be post-transcriptionally processed. Processing of RNA I has also been proposed for the Fst TA system as a mechanism of translation activation^14^, even though the processed transcript has never been isolated. This finding supports the idea that the 5′ UTR of Lpt-encoding RNA might have a role in the regulation of translation process during toxin synthesis. For the Fst system, it has been indeed demonstrated that an helix (UH) and a stem-loop (SL) in the 5′ UTR control the stability of the RNA and its translation^24,25^. In addition, it has also been proposed that a region of RNA I (DR), including the Fst toxin start codon, is sequestered by a complementary region (DR) of the antitoxin RNA II preventing toxin translation^23^.

By mapping the position of DNA bound σ^70^-RNAP, we determined that the predicted promoters are actual promoters and no other strong promoters are present in the TA system DNA region. Furthermore, analysis of the promoter occupancy revealed that the RNA II promoter has a higher affinity than the RNA I promoter, suggesting that the unstable antitoxin is under the control of a stronger promoter. Such a different promoter strength could be due to the particular sequence of the −10 and −35 hexamers but also to the different length of the intervening sequence between the two promoter elements (20 bp and 17 bp for toxin and antitoxin promoters, respectively). When *E*. *coli* was transformed with a vector carrying the Lpt TA system under the control of these natural promoters, growth was not hampered, even when the antitoxin promoter was inactivated, suggesting that the TA system may be regulated by *Lactobacillus* specific factors. However, we systematically observed that after the exponential phase the OD_600_ of cells transformed with an inactivated antitoxin promoter dropped more rapidly than the OD_600_ of cells transformed either with an inactivated toxin promoter or with a *wt* promoter. This behavior may indicate that a toxin concentration threshold must be reached before Lpt can exert its toxic effect.

In this study, we also found that Lpt and its homologous peptides are widespread among *Lactobacillus* genus and in particular in *L*. *casei*, *L*. *paracasei* and *L*. *rhamnosus*. Notably, the distribution of this putative type I TA system is restricted to plasmid DNA because when the entire TA locus was used as a query to search in the nucleotide database, no significant homology was found with bacterial chromosomes. Surprisingly, in a recent multiple approach study^18^, highlighting the wide distribution of type I TA modules in plasmid and chromosome DNA, the Lpt toxin and its homologous peptides were not described.

Lpt homologous peptides encoded by different plasmids show a distribution which seems to be strain-dependent instead of specie-dependent, probably reflecting a possible horizontal gene transfer. In previous studies, type II TA systems encoded by the chromosome of *L*. *rhamnosus* have been proposed as a marker for strains diversity^34^. The synteny analysis of plasmids containing sequences homologous to Lpt reveals that they share a limited region of similarity encompassing Lpt and two neighboring genes potentially involved in plasmid replication. A physical association between genes encoding toxic peptides and genes involved in plasmid replication has been reported also for other TA systems^31^.

Closely genetically related species of the *L*. *casei* group are generally used in commercial and traditional fermented foods. In dairy products their presence is due to the natural occurrence in milk and to the ability of growing in curd and cheese, becoming dominant species in ripening cheese^26^. Ripening represents a crucial step for bacterial growth and survival because different factors, such as salt concentration, pH, moisture, temperature and nutrient starvation, can cause environmental changes leading to stress-induced reactions. Recently, it has been shown that species of *L*. *casei* group can survive in a hostile environment by means of different mechanisms of adaptation^3,35–37^. Plasmids can contribute to this stress tolerance by encoding traits that confer adaptive advantages^4^.

Despite the presence in the literature of many studies concerning the physiological role of TA systems, particularly in pathogenic microorganisms^32,38^, TA system regulation has never been reported for microorganisms grown in food or in food-like environments. In this work, we demonstrate the upregulation of putative toxin and antitoxin RNAs in *L*. *rhamnosus* grown in cheese mimicking conditions. In addition, toxin-encoding RNA was also detected directly in Parmigiano Reggiano cheese ripened either 6 or 12 months. It should be noted that despite *in vitro* RNAP promoter recruitment experiments show that the antitoxin promoter is stronger than the toxin promoter, we find that the ratio between the absolute amount of toxin and antitoxin RNA is in the range 1.6–2.9, in all the experimental conditions analyzed. Although these results do not allow definitive conclusions about the expression regulation of the Lpt peptide, the widespread distribution in the *Lactobacillus* genus of this putative TA system, underlines the importance of plasmid maintenance which, in turn, can affect the composition of the viable bacterial population strictly correlated to technological performance. *In vivo* and *in vitro* experiments aimed to unravel the expression regulation and the mechanism of action of the Lpt toxic peptide are in progress.

## Methods

### Bacterial strains, media and culture conditions

*L*. *rhamnosus* PR1019 and PR1473 were isolated from Parmigiano Reggiano cheese (PR) at 4 and 20 months of ripening, respectively^39^. Strains were identified by 16 S rDNA gene sequencing^40^ and species-specific PCR^41^. Both strains were cultivated in MRS broth (Oxoid) or Cheese Broth (CB) at 30 °C, under anaerobiosis, for 24 or 48 h, respectively. CB is a culture medium that mimics raw-milk long-ripened cheese, prepared according to^42^.

### cDNA-AFLP

*L*. *rhamnosus* PR1473 and PR1019 were grown in MRS or CB medium and the total RNA was extracted at the end of logarithmic phase by using the RNeasy Protect Bacteria Mini Kit (QIAGEN). cDNA synthesis and cDNA-AFLP analysis were carried out as described in^42^ and in Supplementary Methods.

Transcript-derived fragments overexpressed in CB medium were eluted from the gel as described in^43^ and re-amplified using unlabeled selective primers (Table S1). Amplified products were cloned in pGEM vector (Promega) and recombinant plasmids were sequenced on both strands.

### In-silico analysis

NCBI Nucleotide database was searched with the Blastn program^19^ by using the overexpressed cDNA sequence identified by AFLP or the complete Lpt TA system region as a query. Coding region search was conducted with BlastX (nr database) and CD-search (Ver. 3.12) programs, without the filter for low-complexity regions, using the cDNA sequence as a query. Protein homology determination was performed with the HHPRED program^21^ (http://toolkit.tuebingen.mpg.de/hhpred) on the Pfam and PDB databases. The identification of putative promoter sequences was carried out with the BPROM service (http://www.softberry.com), while the prediction of terminator sequences was carried out with the “Arnold finding terminators” web service (http://rna.igmors.u-psud.fr). RNA secondary structures were predicted by using the RNAfold web service (http://rna.tbi.univie.ac.at/cgi-bin/RNAWebSuite/RNAfold.cgi). Sequence alignments were constructed by Clustal Omega^44^ and rendered with the GeneDoc or ESPript3^45^ (http://espript.ibcp.fr) programs.

### Synteny analysis

Analysis of gene order conservation (microsynteny) was performed with the SimpleSinteny web server (http://www.dveltri.com/simplesynteny/) using the protein sequences identified in the pLBPC-2 plasmid as a gene query file and the DNA sequences of 10 Lpt-encoding plasmids as a genome contig file. The Lpt consensus sequence obtained from peptide alignment (without Fst, Fig. 7b) was used in the identification of homologous sequences in all plasmids.

### Sequencing and 5′/3′ RACE-PCR

Plasmid DNA was extracted from *L*. *rhamnosus* PR1019 e PR1473 grown in MRS or CB medium by using Plasmid DNA Extraction Mini Kit (Fisher Molecular Biology). The predicted complete sequence, including all the regulatory regions, was amplified by standard PCR with primers TA-plus and TA-minus (Table S1) designed on the basis of plasmid pLBPC-2 plasmid sequence (GenBank accession number AP01254). The amplified DNA products were sequenced on both strands. Enriched polyadenylated mRNA from PR1019 and PR1473 strains grown in CB medium was used in 5′/3′ RACE-PCR experiments in order to identify the full length sequence of the toxin-encoding RNA (see Supplementary Methods).

### Quantitative reverse transcription PCR

Quantitative reverse transcription PCR (qRT PCR) was carried out on cDNA obtained from RNA of *L*. *rhamnosus* grown in different conditions or from RNA extracted directly from cheese. Total RNA was extracted from *L*. *rhamnosus* PR1473 and PR1019 grown in MRS or CB medium at the end of logarithmic phase by using RNeasy Protect Bacteria Mini Kit. The same procedure was also applied to isolate total RNA from two samples of Parmigiano Reggiano cheese ripened at 6 and 12 months. cDNA was generated from total RNA using the QuantiTect Reverse Transcription Kit (QIAGEN) and random hexamer primers according to the manufacturer’s protocol. qRT PCR was carried out as described in Supplementary Methods.

### Expression of Lpt toxic peptide in *E. coli*

The toxin cDNA sequence comprised between the start codon and the transcription terminator was PCR amplified by using *L*. *rhamnosus* PR1473 plasmid DNA as template and two sequence specific primers: the *Nde*I-tailed Lpt-plus and the *Bam*HI-tailed Lpt-minus (Table S1). The amplification product was cloned into pGEM vector, digested with *Bam*HI and *Nde*I enzymes and subcloned into the expression vector pSRKKm^27^. The recombinant plasmid pSRKKm-lpt was used to transform *E*. *coli* DH10bT1R. Growth assays were carried out in LB medium supplemented with glucose or lactose to a final concentration of 10 mM. *E*. *coli* DH10bT1R transformed with the empty vector pSRKkm was used as a control.

### DNA templates for AFM imaging

pGEM-TA plasmid harboring the entire TA locus was obtained by cloning the 431 bp DNA amplified from *L*. *rhamnosus* PR1019 plasmid DNA using primers TA-plus and TA-minus into pGEM vector. RNA I and RNA II promoter mutants were obtained from pGEM-TA by using QuikChange II Site-Directed Mutagenesis Kit (Agilent). Mutant RNA I and mutant RNA II were obtained by introducing a three-point mutation in the −10 hexamer (TATACT → T**G**T**C**C**C;** TAAAAT → T**G**A**C**A**C**). Mutation fidelity was verified by sequencing.

The 1065 bp DNA templates harboring *wt* RNA I and *wt* RNA II promoters or mutant RNA I and *wt* RNA II promoters or *wt* RNA I and mutant RNA II promoters, were obtained by standard PCR from the corresponding pGEM-TA plasmid with oligonucleotides pGEM-AFM-plus and pGEM-AFM-minus (Table S1). All DNA fragments were gel purified, electroeluted and the DNA concentration was determined by OD_260_ nm. Transcription complexes were assembled and imaged by AFM as described in Supplementary Methods. DNA contour length measurements were performed as described in^46^ and in Supplementary Methods.

### In vivo assays of RNA I and RNA II promoter activity

*E*. *coli* XL1 Blue were transformed with the recombinant plasmid pGEM-TA harbouring the entire Lpt TA locus under the control of *wt* or alternatively inactivated natural promoters. Growth assays were carried out in LB medium and cell growth was monitored by absorbance at 600 nm for twelve hours.

## Electronic supplementary material

Supplementary Information
