# Supplementary material for: Toward the identification of a type I toxin-antitoxin system in the plasmid DNA of dairy Lactobacillus rhamnosus
Source: Sci Rep. 2017 Sep 21;7:12051. doi: 10.1038/s41598-017-12218-5 (PMC5608710; doi:10.1038/s41598-017-12218-5)
Supplement: Supplementary file 1 — Supplementary Information [file 41598_2017_12218_MOESM1_ESM.pdf]

Toward the identification of a type I toxin-antitoxin system in the plasmid DNA of dairy *Lactobacillus rhamnosus*

Claudia Folli, Alessia Levante, Riccardo Percudani, Davide Amidani, Stefania Bottazzi, Alberto Ferrari, Claudio Rivetti, Erasmo Neviani and Camilla Lazzi

Supplementary Methods

<u>cDNA-AFLP</u>

After mRNA enrichment and polyadenylation of RNA transcripts, cDNA was synthesized by reverse transcription (RT) using a biotinylated oligo-dT. A first restriction digestion was performed with *Eco*RI; biotinylated fragments were separated by using streptavidine-coated Dynabeads and further digested with *Mse*I. cDNA fragments were ligated to the specific adaptors (Table S1) and amplified by using the non-selective EcoRI and MseI primers (Table S1). Subsequently, selective amplifications were performed with a *Eco*RI primer labeled with an infrared dye (IRDye™ 700 phosphoramidite), and an unlabeled *Mse*I primer (Table S1). To selectively amplify the expressed genes, three primer combinations were used as follows: DY-EcoRI-AC/MseI-AT, DY-EcoRI-AT/MseI-AC and DY-EcoRI-AT/ MseI-AT (Table S1). cDNA-AFLP fragments were separated by polyacrylamide gel electrophoresis and visualized by Odissey (LI-COR Biosciences).

<u>5'/3' RACE-PCR and DNA sequencing</u>

In 3' RACE-PCR the synthesis of the first cDNA strand was carried out by using the oligo-dT-adaptor primer (anchored polyT, Table S1) and SuperScript II reverse transcriptase (Invitrogen). Touch-down PCR was then employed to amplify the 3' end of cDNA using the forward gene-specific primer 3RACE-plus 3 μM (Table S1) and the reverse primer 3-5RACE-PCR 1 μM (Table S1), corresponding to the adaptor sequence. 5' RACE-PCR was carried out using SMART™ mRNA Amplification Kit (Clontech) following manufacturer's instructions with some changes. Briefly, the synthesis of the first cDNA strand was performed by using the oligo-dT-adaptor primer anchored polyT (Table S1) and SuperScript II reverse transcriptase (Invitrogen), in the presence of the oligo-dG-adaptor primer anchored polyG (Table S1). The cDNA was then amplified by using forward and reverse primers corresponding to adaptor sequences (5RACE PCR primer and 3-5RACE-

PCR primer, Table S1). Finally, the specific cDNA sequence was amplified by using touch-down PCR, the 5RACE PCR primer 1 μM and the reverse gene-specific primer 5RACE-minus 3 μM. The amplification products were then cloned into pGEM vector (Promega) and recombinant plasmids were sequenced on both strands.

Quantitative reverse transcription PCR

qRT PCR was carried out using QuantStudio® 3 (Thermo Fisher Scientific), Power SYBR Green PCR Master Mix (Applied Biosystems) and specific primers as reported in Table S2. The 20 µl PCR reactions were incubated at 95 °C for 10 min, followed by 40 cycles of 95 °C for 15 s and 60 °C for 1 min. Threshold cycle (Ct) data were determined using the default threshold settings and mean Ct values were determined from three PCR replicates.

Absolute quantification of toxin and antitoxin cDNAs from *L.rhamnosus* PR1019 and PR1473 was achieved by using a strain-specific calibration curve of Ct values obtained with increasing amounts of the lpt-containing DNA amplified by PCR with primers TA-plus and TA-minus (Table S1). Slope, efficiency and confidence values of the calibration curve obtained for each specific target and primer combination, are reported in Table S2. ANOVA followed by Tukey's test was applied to compare toxin/antitoxin cDNAs ratios calculated under the different experimental conditions. In the case of Parmigiano Reggiano cheese samples, relative toxin cDNA quantification was calculated using the 2− ΔΔCT method[47], and 16s rRNA was used as internal standard. Statistical significance was determined by means of the Student's t-test for pairwise comparisons. All statistical analysis were performed using IBM SPSS v.23.

Transcription complex assembly and AFM imaging

Promoter complexes were obtained by mixing 25 nM DNA with 70 nM *E.coli* RNAP holoenzyme (New England Biolabs) in transcription buffer (20 mM Tris–HCl pH 7.9, 50 mM KCl, 5 mM $MgCl_2$, 1 mM DTT) and incubated for 30 min at 37 °C. The reaction was diluted 10X in deposition buffer (4 mM HEPES pH 7.4, 10 mM NaCl, 2 mM $MgCl_2$) and deposited onto freshly-cleaved mica for 90 seconds. Afterwards, the mica disk was rinsed with MilliQ water and dried with a gentle nitrogen flow. AFM imaging was performed in air with the tapping mode using a Nanoscope IIIA microscope (Veeco) equipped with E scanner and NSC14/Al

BS commercial silicon cantilevers (MikroMasch). Images of 512×512 pixels were collected with a scan size of 2 µm at a scan rate of 2.5 lines per second.

DNA contour length measurements were performed as described in[47] using the following contour length estimator: L = (0.963ne + 1.362no) x S/W, where “ne” and “no” are the number of even and odd chain codes respectively, S is the image scan size, W is the image width in pixels. Position of the DNA bound RNAP was manually selected by clicking with the mouse the center of the protein globular feature. The DNA contour length from the protein to the nearest end was defined short-arm while the DNA contour length from the protein to the farther end was defined long-arm.

Supplementary Table 1. List of oligonucleotides used in this study

Name	Sequence (5'-3')	Application
EcoRI	GACTGCGTACCAATTC	non-selective primer for cDNA-AFLP
MseI	GATGAGTCCTGAGTAA	non-selective primer for cDNA-AFLP
5'DY-EcoRI-AT	GACTGCGTACCAATTCAT	selective labelled primer for cDNA-AFLP
5'DY-EcoRI-AC	GACTGCGTACCAATTCAC	selective labelled primer for cDNA-AFLP
MseI-AT	GATGAGTCCTGAGTAAAT	selective primer for cDNA-AFLP
MseI-AC	GATGAGTCCTGAGTAAAC	selective primer for cDNA-AFLP
EcoRI-plus	CTCGTAGACTGCGTACC	adaptor for cDNA-AFLP
EcoRI-minus	AATTGGTACGCAGTCTAC	adaptor for cDNA-AFLP
MseI-plus	GACGATGAGTCCTGAG	adaptor for cDNA-AFLP
MseI-minus	TACTCAGGACTCAT	adaptor for cDNA-AFLP
TA-plus	GTTGAAATTCACAATAACTTTGCAC	primer for PCR and sequencing of TA system
TA-minus	CCATAACGAAGTTTTGACTATTTTTG	primer for PCR and sequencing of TA system
anchored polyT	ACAACTTTGTACAAGAAAGTTGGGT ACT(30)VN	oligo-dT-adaptor primer for 3' and 5'RACE
3RACE-plus	AGTTCATGAATTCATTCGATAAAGC	sequence-specific primer for 3' RACE and RNA I qRT PCR
5RACE-minus	GTTATCCAGAGAGTATTTCAACA	sequence-specific primer for 5' RACE and RNA I qRT PCR
anchored polyG	GGACAACTTTGTACAAAAAAGTTGGA GAGGGCGGG	oligo-dG-adaptor primer for 5' RACE
5RACE PCR	TCGTCGGGGACAACTTTGTACAAAAAA GTTGG	primer corresponding to adaptor sequence for 5' RACE
3-5RACE-PCR	GGCGGCCGCACAACTTTGTACAAGAA AGTTGGGT	primer corresponding to adaptor sequence for 3'and 5'RACE
Lpt-plus	CATATGAATTCATTCGATAAAGCGA	primer for Lpt cDNA cloning
Lpt-minus	GGATCCAAGCCATCATCTCCG	primer for Lpt cDNA cloning
RNAII 1019-plus	CGAATGAATTTATGAACATCACA	primer for RNA II qRT PCR
RNAII 1019-minus	CATGCAGCTTGCGGACTA	primer for RNA II qRT PCR
RNAII 1473-plus	GAATTCATGAACATCACAAACCCGT	primer for RNA II qRT PCR
RNAII 1473-minus	AAAACCCGTGCAGCTTGCG	primer for RNA II qRT PCR
pGEM-AFM-plus	CGGTGCGGGCCTCTTCGC	primer for PCR of AFM fragment
pGEM-AFM minus	TCTGTGGATAACCGTATTACCG	primer for PCR of AFM fragment
RNAImut-plus	GGCGTAGCGACTAAACTGGGACACCA AAAAGCAGTAAACACC	primer for mutagenesis of RNA I -10 promoter
RNAImut-minus	GGTGTTTACTGCTTTTTGGTGTCCCAGT TTAGTCGCTACGCC	primer for mutagenesis of RNA I -10 promoter
RNAIImut-plus	GCAACTGGTTATAATCTATGCTGACAC GAACCTAATCGAATG	primer for mutagenesis of RNA II -10 promoter
RNAIImut-minus	CATTCGATTAGGTTCGTGTCAGCATAG ATTATAACCAGTTGC	primer for mutagenesis of RNA II -10 promoter

Supplementary Table 2. Calibration curve parameters for qRT PCR

Target	Forward primer	Reverse primer	Strain	Slope	Efficiency (E)[1]	R^2
RNA I	3RACE-plus	5RACE-minus	1019	-3.34	1.99	0.9998
RNA I	3RACE-plus	5RACE-minus	1473	-3.08	2.25	0.9971
RNA II	RNAII 1019-plus	RNAII 1019-minus	1019	-3.55	1.82	0.9993
RNA II	RNAII 1473-plus	RNAII 1473-minus	1473	-3.10	2.22	0.9969

[1] Primer efficiency is calculated as follows: $E = 10^{-1/slope}$, as described in Pfaffl M.W., 2001, Nucleic Acids Research, 29, e45

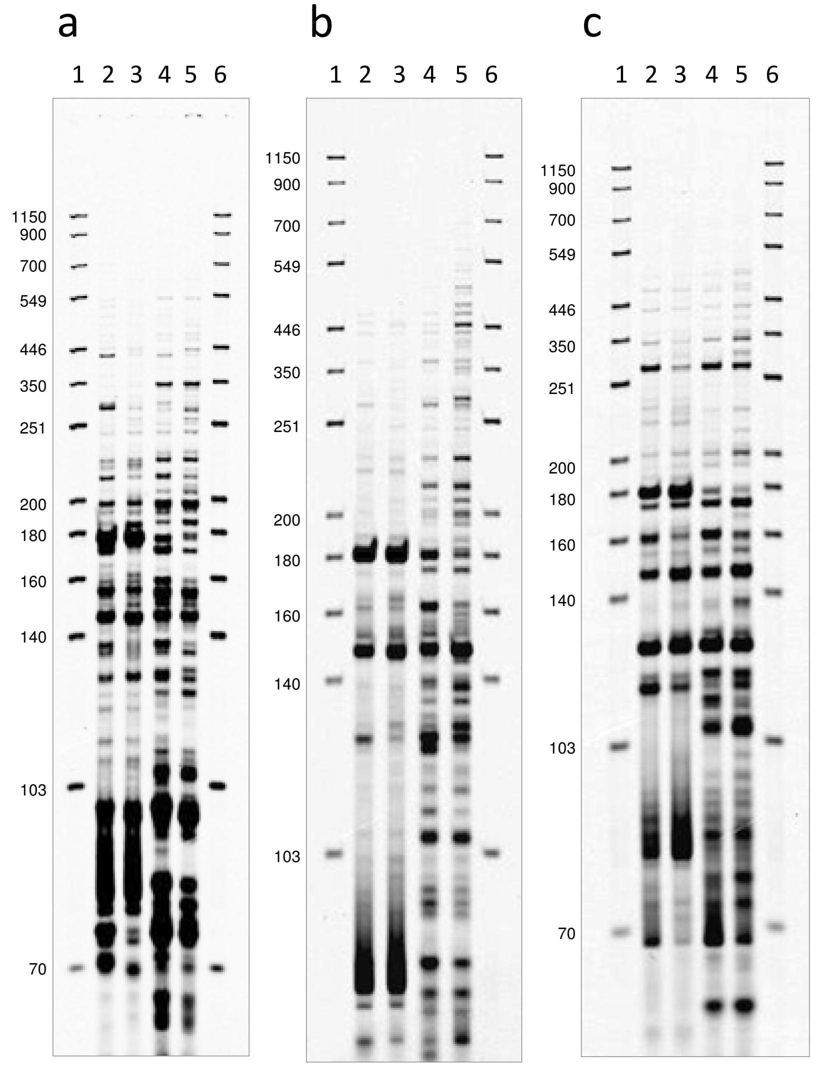

Supplementary Figure 1. Full length electrophoretic patterns of cDNA-AFLP experiments conducted on *L. rhamnosus* strains

a) cDNA-AFLP fingerprinting analysis conducted with the primer combination EcoRI-AC/MseI-AT. Lane 1, IRDye700 sizing standard; lane 2, PR1019 in MRS medium; lane 3, PR1019 in CB medium; lane 4, PR1473 in MRS medium; lane 5, PR1473 in CB medium; lane 6, IRDye700 sizing standard.

b) cDNA-AFLP fingerprinting analysis conducted with the primer combination EcoRI-AT/MseI-AC. Lane 1, IRDye700 sizing standard; lane 2, PR1019 in MRS medium; lane 3, PR1019 in CB medium; lane 4, PR1473 in MRS medium; lane 5, PR1473 in CB medium; lane 6, IRDye700 sizing standard.

c) cDNA-AFLP fingerprinting analysis conducted with the primer combination EcoRI-AT/MseI-AT. Lane 1, IRDye700 sizing standard; lane 2, PR1019 in MRS medium; lane 3, PR1019 in CB medium; lane 4, PR1473 in MRS medium; lane 5, PR1473 in CB medium; lane 6, IRDye700 sizing standard.

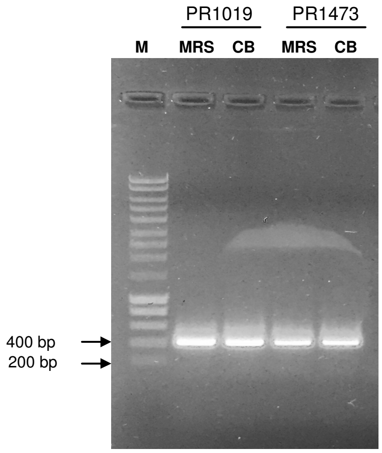

Supplementary Figure 2. PCR-amplification of Lpt TA locus from *L. rhamnosus* strains
Agarose gel electrophoresis of the amplification products obtained by standard PCR using plasmid DNA extracted from *L. rhamnosus* PR1473 and PR1019 grown in MRS or CB medium. M: marker SMART ladder (Biosense).

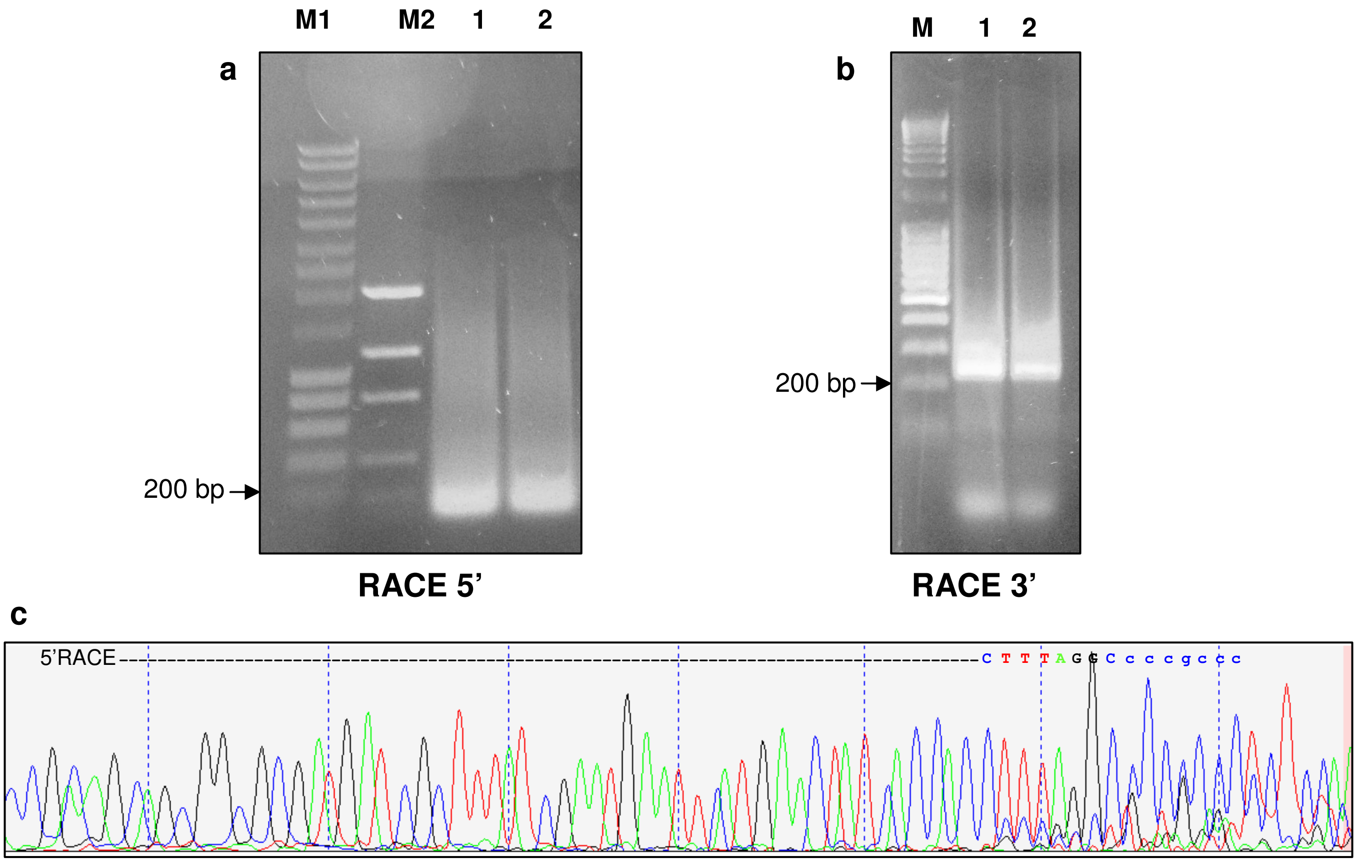

Supplementary Figure 3. 5'/3' RACE experiments

a) Agarose gel electrophoresis of the amplification products of 5' RACE experiments (lanes 1 and 2); M1, marker SMART ladder (Biosense); M2, marker Low DNA mass ladder (Invitrogen).

b) Agarose gel electrophoresis of the amplification products of 3' RACE experiments (lanes 1 and 2); M, MassRuler DNA Ladder Mix (Thermo Fisher Scientific);

c) *L. rhamnosus* PR1473 sequencing profile of the amplified fragment obtained by 5' RACE experiments. Uppercase characters correspond to the 5' sequence of RNAI molecule and lowercase characters indicate the sequence of the primer.

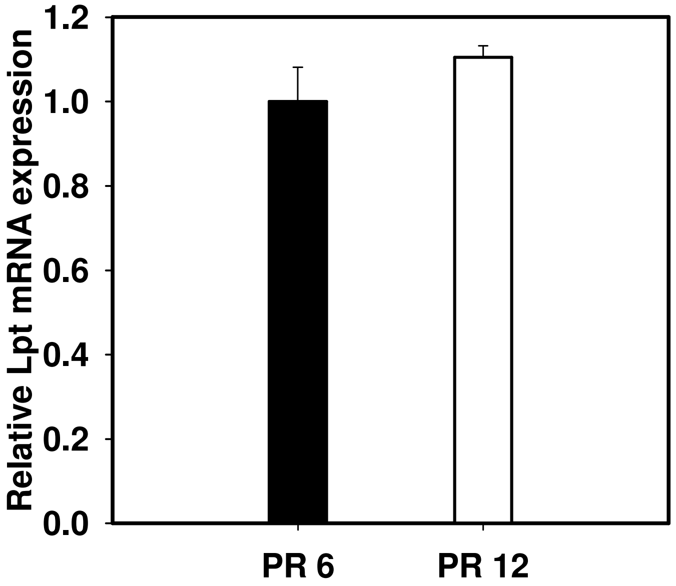

Supplementary Figure 4. Detection of RNA I in Parmigiano Reggiano cheese
Relative Lpt mRNA expression detected by qRT PCR in 12 month ripened cheese (PR 12, white bar) compared to 6 month ripened cheese (PR 6, black bar). The error bars represent the standard deviation of the mean value from three independent experiments.

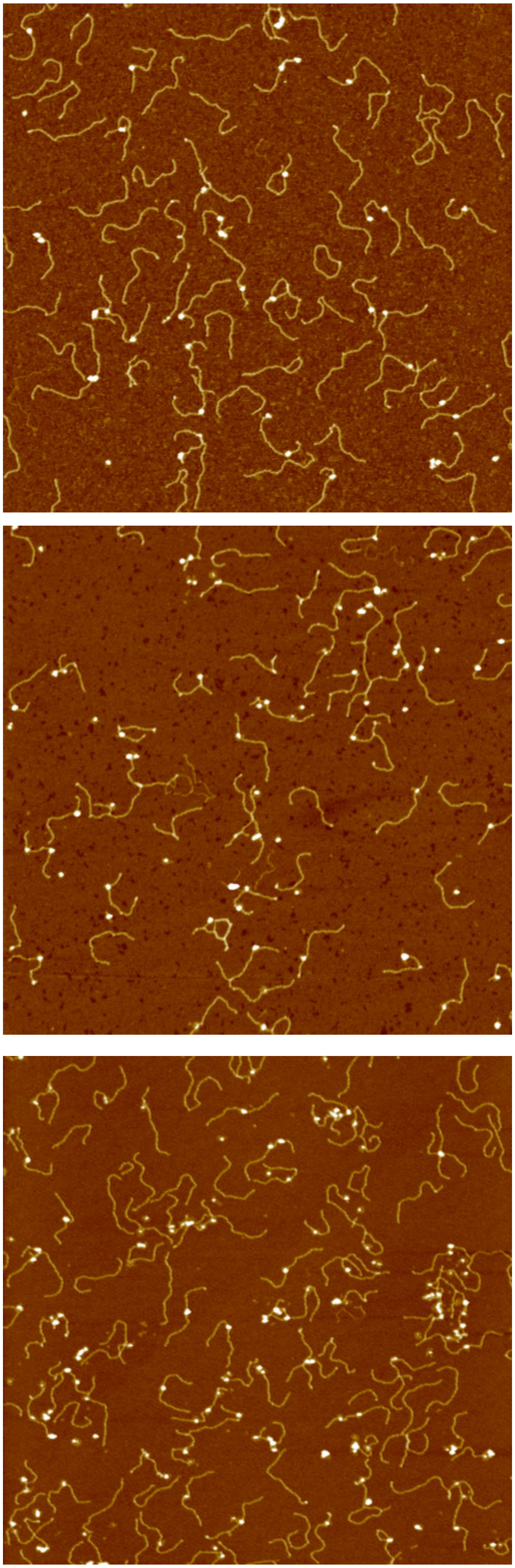

Supplementary Figure 5. Raw AFM images of promoter complexes assembled on a 1065 bp DNA template harbouring a) *wt*RNA I and *wt*RNA II promoters; b) mutated RNA I and *wt*RNA II promoters; c) *wt*RNA I and mutated RNA II promoters. Scan size: 2 mm.

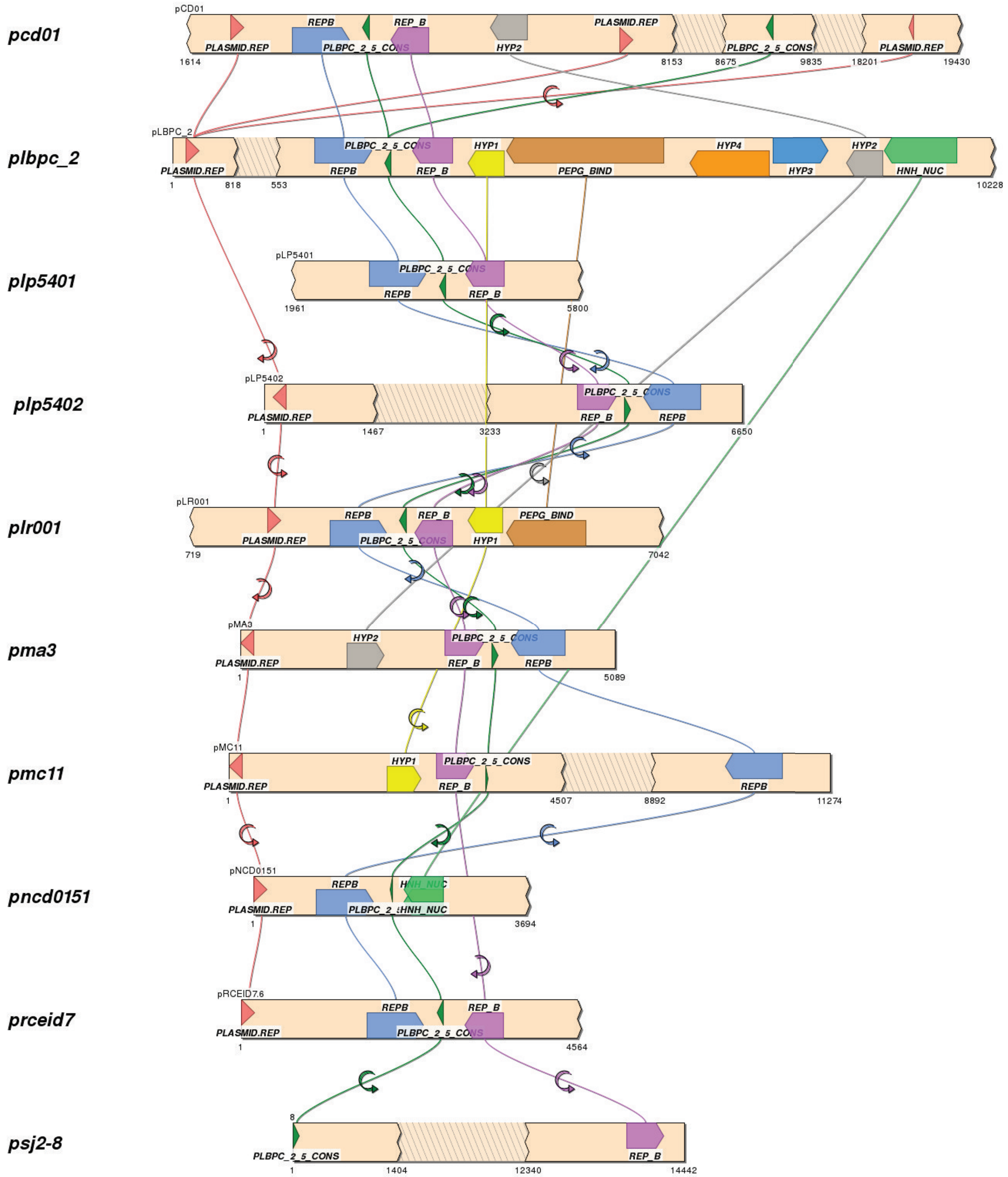

Supplementary Figure 6. Sinteny analyses of plasmids containing Lpt TA locus homologs
